# Designing Inorganic–Organic Dual-Acid Deep Eutectic Solvents for Synergistically Enhanced Extractive and Oxidative Desulfurization

**DOI:** 10.3390/molecules28237743

**Published:** 2023-11-24

**Authors:** Dongao Zhu, Lixian Xu, Beibei Zhang, Linhua Zhu, Jing He, Hongping Li, Huaming Li, Wei Jiang

**Affiliations:** 1 Institute for Energy Research, School of Chemistry and Chemical Engineering, Jiangsu University, Zhenjiang 212013, China; zda12281025@163.com (D.Z.); 18260630971@163.com (L.X.); hej@ujs.edu.cn (J.H.); hongpingli@ujs.edu.cn (H.L.); lhm@ujs.edu.cn (H.L.); 2 School of Chemistry and Chemical Engineering, Yancheng Institute of Technology, Yancheng 224051, China; yizhou1118@163.com; 3 Key Laboratory of Water Pollution Treatment and Resource Reuse of Hainan Province, College of Chemistry and Chemical Engineering, Hainan Normal University, Haikou 571158, China; zhulinhua@hainnu.edu.cn

**Keywords:** dual-acid deep eutectic solvent, inorganic and organic acids, synergistic effect, oxidative desulfurization, mechanism

## Abstract

Acidic deep eutectic solvents (DESs) have been considered desirable extractants and catalysts for desulfurization. However, their hydrogen bond donors (HBDs) are usually sole organic acids, which are not conducive to efficient green catalysis. Herein, a novel inorganic–organic dual-acid DES (DADES) was reported for efficient extractive and oxidative desulfurization. Benefiting from the physical interaction among the three components in a DADES, a transparent homogeneous liquid can be obtained even though inorganic acid (boric acid, BA) and organic acid (acetic acid, AA) can be immiscible. Furthermore, the dual-acid HBD can increase the acidity of the DADES and reduce its viscosity, accelerating its mass transfer efficiency and enhancing its catalytic activity. With 1-butyl-3-methylimidazolium chloride ([Bmim]Cl) as the hydrogen bond acceptor, [Bmim]Cl/BA/0.3AA effectively activated hydrogen peroxide and achieved sulfur removal of 96.6% at 40 °C. Furthermore, the universality of the synergistic effect in various DADESs was confirmed by the modulation of the types of organic acids. This study not only motivates the construction of more intriguing novel DESs based on the DADES concept but also highlights their potential in clean fuel production.

## 1. Introduction

Under the trend of rapidly increasing energy consumption, the widespread burning of S-containing compounds in fuels increases hazardous sulfur oxide (SO_x_) emissions, forming acid rain and severely damaging the environmental equilibrium [1]. It directly leads to vegetation destruction, industrial equipment corrosion, and respiratory diseases [2,3]. Consequently, global legislation was progressively tightened to reduce the S-content of fossil fuels below 10 ppm [4,5]. Extensive research has concentrated on removing S-containing compounds to produce low-sulfur fuel. Hydrogen desulfurization (HDS), as a well-established desulfurization technology, remains the dominant technology for industrial applications [6]. However, HDS is an energy-intensive process demanding high temperatures (320–380 °C) and pressures (3–10 MPa) [7,8]. Moreover, several refractory aromatic sulfides and derivatives are difficult to eliminate effectively by HDS technology due to inefficient hydrogenation, which requires more stringent operating conditions [9,10]. Several targeted complementary technologies have emerged, including adsorptive desulfurization [11], extractive desulfurization (EDS) [12], and oxidative desulfurization (ODS) [13] etc. Extractive and oxidative desulfurization (EODS) technology is regarded as one of the most promising technologies due to its exceptional ability to remove aromatic thiophenes under moderate operating conditions [14,15]. Hydrogen peroxide (H_2_O_2_) as an oxidant has attracted wide attention since it is affordable, widely accessible, and has no hazardous by-products [16]. It is noteworthy that the ODS process requires an effective catalyst to ensure the sufficient oxidative activity of H_2_O_2_ [17]. In addition, for some solid catalytic systems, the oxidative product may be retained in its oil phase due to inadequate adsorption capability, which requires the addition of an extra extractant to achieve desulfurization [18]. Hence, designing a suitable catalytic system with strong extraction/adsorption and catalysis is a significant issue.

Defined as eutectic mixtures of two or three biocompatible and non-toxic substances, DESs have a strong capability to form intermolecular hydrogen bonds, enabling almost 100% of raw material utilization [19]. Being analogues of ionic liquids (ILs), DESs exhibit several additional advantages, like a low price, easy preparation, high atomic utilization, biodegradability, and flexible adjustability [20,21]. Consequently, the emergence of DESs has attracted considerable attention in many fields, including electrochemistry [22], biochemistry [23], separation processes [24], and catalytic reactions [25]. Due to their designability and superior surface-active advantage, DESs offer a novel possibility to enhance desulfurization. Initially, DESs were confirmed to have significant potential for EDS due to their ability to form intermolecular interactions with sulfur compounds [12]. However, to meet the rigorous S-concentration standard, multistage extraction is required [26]. Subsequently, DESs were combined with other catalysts to achieve deep desulfurization by the EODS process, with DESs acting as extractants [27]. Selecting suitable HBDs and hydrogen bond acceptors (HBAs) of DESs is essential to advance the EODS process [28,29]. Several studies and theoretical calculations have confirmed the indispensability of acidic groups for the ODS reaction, directly influencing their catalytic activity [30,31,32]. Moreover, a new type of ternary DESs using inorganic acids as HBDs was reported to achieve deep desulfurization by our group, which was formed by choline chloride, polyethylene glycol, and boric acid [33]. Nevertheless, the weak acid and high viscosity of the ternary DES ChCl/1.5BA/PEG limited its EODS performance.

Herein, boric acid (BA) was combined with several organic acids, such as acetic acid (AA), propionic acid, and oxalic acid, respectively, as the HBDs to produce the DADES. 1-Butyl-3-methylimidazolium chloride ([Bmim]Cl) was chosen as the primary HBA to promote efficient mass transfer [34,35]. Meanwhile, the presence of [Bmim]Cl was used as a bridge to facilitate the mutual solubility of inorganic and organic acids. Through an investigation of their structure–activity relationship, the optimal molar ratio of BA and AA in [Bmim]Cl/BA/0.3AA was determined. Due to the decreased viscosity and enhanced acidity by dual-acid HBDs, [Bmim]Cl/BA/0.3AA reached a sulfur removal of 96.6% within 90 min at 40 °C, exhibiting superior activity to the binary DESs, [Bmim]Cl/BA (75.0%) and [Bmim]Cl/0.3AA (21.9%), containing a single inorganic or organic acid. Moreover, the recyclability and stability of [Bmim]Cl/BA/0.3AA were confirmed, which could be regenerated by diethyl ether washing. This work aims to realize the possibility of co-activation of inorganic and organic acids using the DADES strategy, expanding thoughts for the design of a novel DES.

## 2. Results and Discussion

### 2.1. Screening of DESs

**Figure 1:**
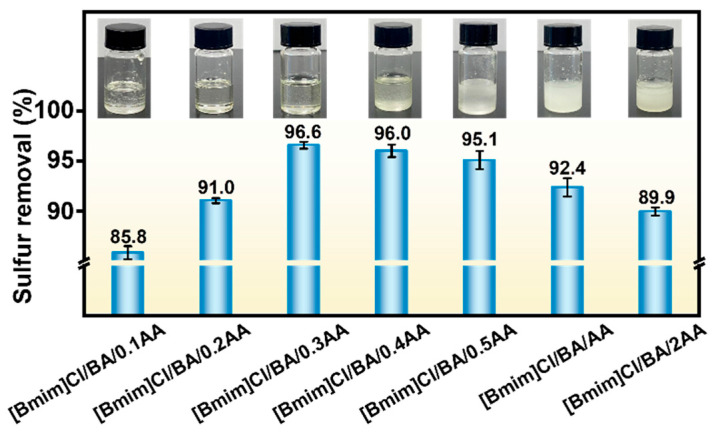
Desulfurization efficiency of DADESs with various AA contents. Experimental conditions: Model oil = 5 mL, O/S = 5, m (DES) = 1.5 g, t = 90 min, T = 40 °C.

Firstly, BA and AA were mixed and stirred in a round-bottomed flask at 60 °C, but they did not dissolve in each other, illustrating the limited mutual solubility between the inorganic and organic acids (Figure S1). Interestingly, the introduction of [Bmim]Cl allowed for the preparation of homogeneous and transparent liquids, making it possible to synthesize the inorganic–organic DADES system. The structure–activity relationship of the DADES was then analyzed by regulating the AA content in the DADES, [Bmim]Cl/BA/nAA. A series of [Bmim]Cl/BA/nAA DADESs was prepared at 60 °C. The state of the DADESs after cooling to room temperature is displayed in Figure 1. [Bmim]Cl/BA/nAA with an AA molar ratio of 0.3 or less remained clear and transparent. However, white precipitates were observed in the [Bmim]Cl/BA/nAA (*n* > 0.3). As the AA content increased, more white solids precipitated over a short period. It is speculated that the addition of excess AA competed with BA for the opportunity to form hydrogen bonds with [Bmim]Cl, resulting in the above DADESs exhibiting poor stability. Therefore, due to the low mutual solubility of BA and AA, the precipitated white solid could be further identified as BA. Notably, EODS performance gradually decreased with the molar ratio of AA being higher than 0.3, which is related to the reduced stability of DADESs. Consequently, [Bmim]Cl/BA/0.3AA was finally selected for the subsequent investigations.

According to the mutual solubility test, the FT-IR and ^1^H NMR spectra of the oil and [Bmim]Cl/BA/0.3AA showed no significant changes before and after mixing, demonstrating that the DADES does not cause contamination (Figure S2). Thus, the prepared DADES can be used as an extractant and catalyst for an EODS reaction. To evaluate its desulfurization capability, the prepared DADES and individual components were mixed with the sulfur-containing model oil, respectively. After reaching extraction equilibrium, a specified amount of H_2_O_2_ was dissolved to initiate the EODS reaction (Figure S3). As listed in Table 1, the desulfurization efficiencies of BA, AA, and [Bmim]Cl were 3.6%, 2.3%, and 13.1%, respectively. With [Bmim]Cl/BA/0.3AA as the extractant and catalyst, the EDS and EODS efficiencies increased significantly to 22.0% and 96.6%, respectively. Moreover, the desulfurization performance of the binary DESs was also evaluated. [Bmim]Cl/BA achieved extraction and oxidative desulfurization rates of 9.8% and 75.0%, respectively, while the sulfur removal of [Bmim]Cl/AA was only 21.9%, demonstrating the superiority of BA as an active catalytic center [33]. These results prove the advantage of the dual-acid synergistic effect in the EODS process, which will be investigated in detail from the modification of viscosity and acidity.

**Figure 2:**
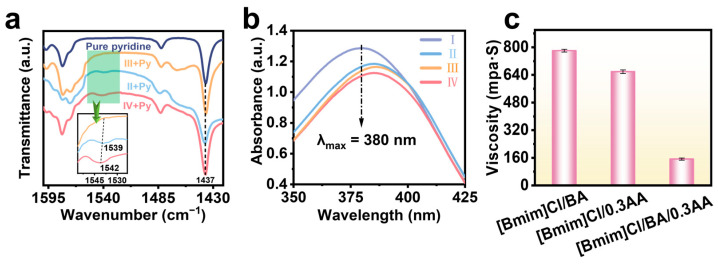
(**a**) FT-IR spectra of (II) [Bmim]Cl/BA, (III) [Bmim]Cl/0.3AA, and (IV) [Bmim]Cl/BA/0.3AA with pyridine, and (**b**) UV–Vis of (I) 4-nitroaniline in (II) [Bmim]Cl/BA, (III) [Bmim]Cl/0.3AA, and (IV) [Bmim]Cl/BA/0.3AA, respectively. (**c**) The viscosity of different DESs at 40 °C.

Consequently, we hypothesized that the synergistic effect observed in the DADES was closely related to its viscosity and acidity, which will be investigated in detail. The acidity of the prepared DESs was determined by employing FT-IR spectra with pyridine as a probe molecule. As shown in Figure 2a, the peak at 1437 cm^-1^ is related to the pure pyridine. There were almost no new absorption peaks in the mixture of [Bmim]Cl/0.3AA and pyridine, indicating slight Brønsted acidity due to its low acid content in [Bmim]Cl/0.3AA. After [Bmim]Cl/BA was mixed with pyridine, a new characteristic peak at 1539 cm^−1^ was observed, indicating it contained Brønsted acidity (Figure 2b) [36]. Of note is the characteristic peak of Brønsted acidity in the [Bmim]Cl/BA/0.3AA blue shifted from 1539 cm^−1^ to 1542 cm^−1^, suggesting an increase in acidity [37]. Furthermore, the Hammett acidity function method was employed for accurate quantitative analysis of acidity differences in the DESs [38]. A UV–Vis spectrophotometer (Kyoto City, Japan) was used to detect the changes in absorbance after mixing 4-nitroaniline aqueous solution (2.9 × 10^−3^ mol/L) with different DESs, and the Hammett function (*H*_0_) was defined by Equation (1).

(1) $$H_{0}=pK{\left(In\right)}_{aq}+\log\left(\frac{\left[In\right]}{\left[{InH}^{+}\right]}\right)$$

where p*K*(In)_aq_ = (0.99) is the dissociation constant of the base indicator. [In] and [InH^+^] represent the concentrations of the unprotonated and protonated indicators of the solution, respectively. As shown in Figure 2b, the maximum absorption wavelength (λ_max_) of 4-nitroaniline is 380 nm, with an associated absorbance (A_max_) of 1.323. Compared to binary DESs, the A_max_ of the indicator decreased most distinctly after dissolution in the [Bmim]Cl/BA/0.3AA, implying a higher acidity. Moreover, the lower the *H_0_* value, the stronger the acidic DES, as shown in Table 2. This acidity was followed by [Bmim]Cl/BA (2.17) < [Bmim]Cl/0.3AA (2.11) < [Bmim]Cl/BA/0.3AA (1.97), which is in agreement with the results of the FT-IR. Activation of H_2_O_2_ was facilitated by increasing the acidity of the DADES, resulting in higher EODS performance. Figure 2c provides the analyzed viscosity for all the DESs. The viscosity of [Bmim]Cl/BA/0.3AA (152.8 mPa·S) was distinctly lower than [Bmim]Cl/BA (780.6 mPa·S) and [Bmim]Cl/0.3AA (658.6 mPa·S), which facilitated its efficient mass transfer, providing a suitable environment for the EODS reaction.

**Figure 3:**
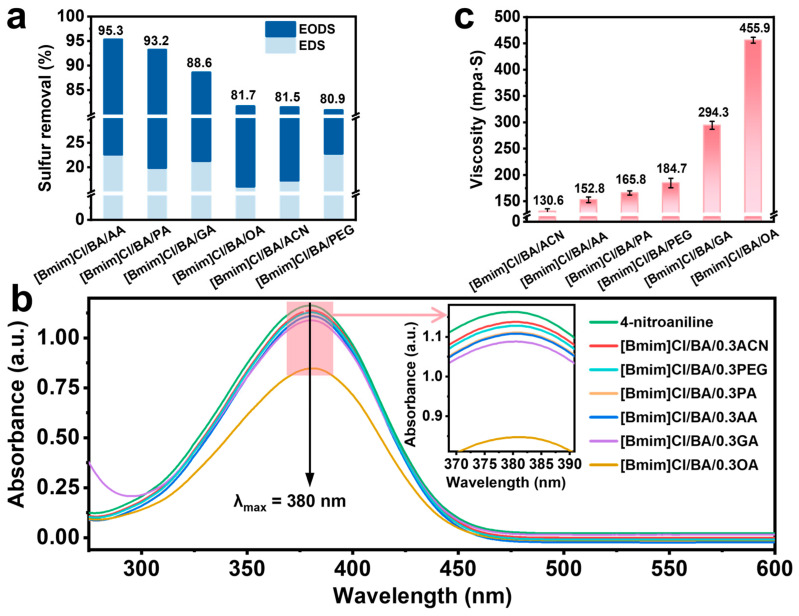
(**a**) Sulfur removal of DESs with different compositions of molar ratios, (**b**) UV–Vis of 4-nitroaniline in various DADESs, and (**c**) viscosity of different DADESs at 40 °C. Experimental conditions: Model oil = 5 mL, O/S = 5, m (DES) = 1.5 g, t = 60 min, T = 40 °C.

Furthermore, PEG and ACN were introduced to prepare ternary DESs for comparative tests. In Figure 3a, sulfur removal of [Bmim]Cl/BA/0.3PEG and [Bmim]Cl/BA/0.3ACN was 80.9% and 81.5%, respectively. Despite the low viscosity of the two, they still exhibited lower activity than [Bmim]Cl/BA/0.3AA, which might be attributed to the neutral and weakly basic nature of PEG and ACN, respectively, demonstrating the advantages of dual-acid HBDs in DADESs. To further confirm the universality of the DADES strategy, various carboxylic acids, including PA, OA, and GA, were introduced as the second acid HBD. In comparison to [Bmim]Cl/BA, with a sulfur removal of 75.0%, the desulfurization efficiency of all DADESs was enhanced (Figure 3a). Although [Bmim]Cl/BA/0.3GA and [Bmim]Cl/BA/0.3OA showed higher acidities than [Bmim]Cl/BA/0.3PA and [Bmim]Cl/BA/0.3AA (Figure 3b and Table S1), the former DADESs showed lower sulfur removal than the latter, which was also related to the influence of viscosity. Therefore, the development of a DADES with high catalytic performance requires comprehensive considerations, including acidity and viscosity.

### 2.2. Characterization of the DADES [Bmim]Cl/BA/0.3AA

**Figure 4:**
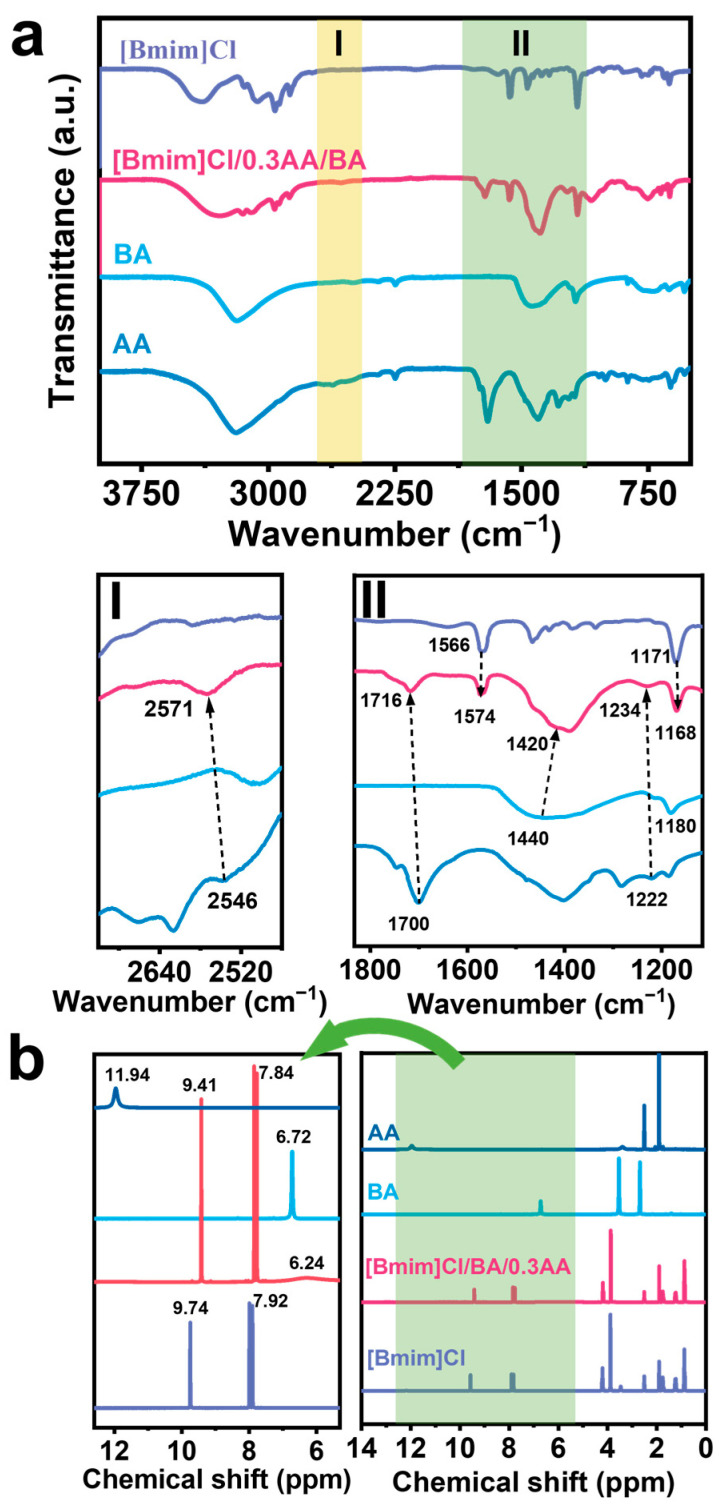
(**a**) FT-IR and (**b**) ^1^H NMR spectra of raw materials and [Bmim]Cl/BA/0.3AA.

The chemical structure of raw materials and intermolecular interactions in [Bmim]Cl/BA/0.3AA were investigated by FT-IR spectra. In Figure 4a, the bending vibration of O–H in BA appeared at 1180 cm^−1^, which was hardly observed in the DADES. Meanwhile, the stretching vibration of the B–O red-shifted from 1440 cm^−1^ to 1420 cm^−1^ with weaker intensity. Such an observation may be related to hydrogen bond interaction [33]. The characteristic peak at 2546 cm^−1^ of the red-shifted to 2571 cm^−1^, resulting in an increase in the dipole moment and a reduction in the force constant in the O–H bond in AA [29]. Moreover, it is noteworthy that the stretching vibration of C=O at 1700 cm^−1^ and the C–O band at 1222 cm^−1^ exhibited a blue shift to 1716 cm^−1^ and 1234 cm^−1^, respectively, indicating that the intermolecular force of AA could have been influenced by H-bonding interaction. Furthermore, [Bmim]Cl revealed two peaks at 1566 cm^−1^ and 1171 cm^−1^, which correspond to the stretching vibration of C=N on the imidazole ring and the in-plane bending vibration of C–H, respectively [39]. They shifted to 1574 cm^−1^ and 1168 cm^−1^, respectively, implying the possible complex H-bonding interaction in [Bmim]Cl/BA/0.3AA.

The nature of H-bonding interaction in [Bmim]Cl/BA/0.3AA was further explored by the ^1^H NMR spectra. In Figure 4b, the hydrogen signals of BA and AA were detected at 6.72 and 11.94 ppm, respectively. After the DADES was formed, the hydrogen signal of BA upshifted to a high electric field at 6.24 ppm, and the intensity was broadened and weakened more significantly than the [Bmim]Cl/BA (Figure S4). Simultaneously, the hydrogen signal of AA at 11.94 ppm disappeared. In addition, the hydrogen signals of [Bmim]Cl in the imidazole ring appeared at 9.74 ppm and 7.92 ppm [40], which downshifted to 9.41 ppm and 7.84 ppm, respectively. It could be speculated that stronger and more complex H-bonding interactions exist in [Bmim]Cl/BA/0.3AA [41].

### 2.3. Evaluation of Desulfurization Performance

**Figure 5:**
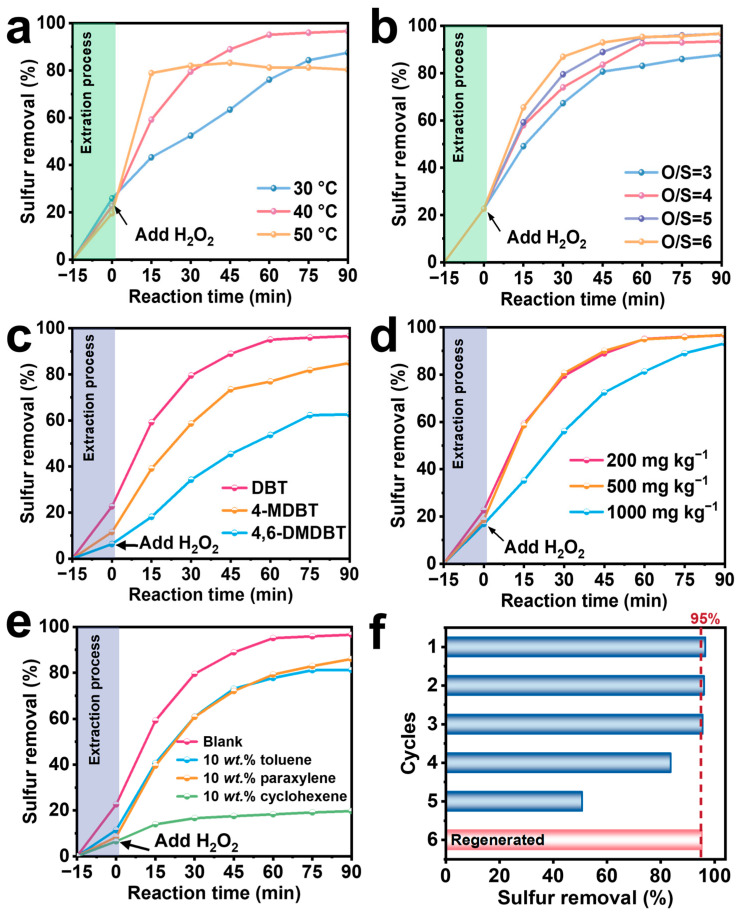
Influence of different (**a**) reaction temperatures, (**b**) O/S molar ratios, (**c**) sulfur compounds, (**d**) initial sulfur content, and (**e**) other compounds in real fuels. (**f**) Evaluation of recyclability of [Bmim]Cl/BA/0.3AA. Experimental conditions: Model oil = 5 mL, T = 40 °C (except a), O/S = 5 (except b), m (DES) = 1.5 g, t = 90 min.

The reaction temperature is a critical parameter that is closely related to desulfurization efficiency and energy consumption. As detected in Figure 5a, the desulfurization efficiency increased from 87.5% to 96.6% after the temperature increased from 30 °C to 40 °C, mainly due to the increasing probability of effective molecular collisions. Simultaneously, the reduced viscosity of [Bmim]Cl/BA/0.3AA with increasing temperature resulted in a high mass transfer efficiency to accelerate reaction efficiency (Figure S5). However, the sulfur removal dropped slightly to 83.2% at 50 °C despite the rapidly accelerated initial period. Except for the unproductive decomposition of H_2_O_2_ at excessive temperatures, the reduced EDS efficiency (from 22.6% to 17.5%) also accounts for the decline in EODS efficiency [42]. Consequently, 40 °C was identified as the optimal temperature.

The dosage of oxidant directly impacts the EODS performance of a DADES. Hence, the effects of different O/S molar ratios (3, 4, 5, and 6) were analyzed. In Figure 5b, the desulfurization efficiencies reached 87.8%, 93.5%, and 96.6% within 90 min at O/S of 3, 4, and 5, respectively. The formation of much more active intermediates was responsible for the progressive improvement in catalytic performance as the dosage increased. However, the improvement in desulfurization capability was insufficient as the amount of H_2_O_2_ continued to increase. Thus, the O/S of 5 is considered the optimal choice.

Model oils containing alkyl-substituted dibenzothiophene compounds were used to further explore the desulfurization capacity of [Bmim]Cl/BA/0.3AA. As illustrated in Figure 5c, sulfur removal for DBT, 4-MDBT, and 4,6-DMDBT was 96.6%, 84.9%, and 62.6%, respectively. Based on the electrophilic addition mechanism, the reactivity of sulfur compounds would be boosted as the electron density of the S atoms increased [43]. However, despite the electron cloud density observed in 4-DMDBT (5.759) and 4,6-DMDBT (5.760) being higher than that of DBT (5.758) [44], the steric hindrance imposed by their methyl substituents restricts the accessibility of S-atoms to their active sites [45]. Consequently, the reactive ability of 4-MDBT and 4,6-DMDBT is reduced, increasing the difficulty of the EODS process.

The desulfurization performance of [Bmim]Cl/BA/0.3AA for model oil with different initial S-contents was evaluated. As shown in Figure 5d, deep desulfurization was achieved for both initial S-contents of 200 mg kg^−1^ and 500 mg kg^−1^ within 90 min at 40 °C, demonstrating the excellent catalytic performance of [Bmim]Cl/BA/0.3AA. While for the oil with an S-content of 1000 mg kg^−1^, the sulfur removal was 93.2% under the same reaction conditions. This is presumably due to the fact that more H_2_O_2_ is used for a 1000 mg kg^−1^ model oil, resulting in more H_2_O being introduced into the system, which might inhibit the desulfurization process. To confirm the above speculation, the EODS reaction was implemented with different mass concentrations of H_2_O_2_ as an oxidant. As shown in Figure S6, the EODS efficiency decreased slightly from 96.6% to 93.9%, with the H_2_O_2_ content reducing from 30 wt.% to 15 wt.%. After adding 5 wt.% of H_2_O_2_ to the reaction system, the desulfurization efficiency was sharply reduced to 45.7%. Owing to the decreased concentration of H_2_O_2_, more water was introduced into the reaction system. This not only weakened the extractive capacity of [Bmim]Cl/BA/0.3AA but also decreased its acidity, reducing the final EODS efficiency. Therefore, a less aqueous environment is more conducive to the desulfurization reaction using a DADES as a catalyst.

Furthermore, the other major components, aromatics and olefins, in actual diesel fuel may interfere with the desulfurization process [18]. Therefore, toluene (10 wt.%), paraxylene (10 wt.%), and cyclohexene (10 wt.%), as typical aromatic hydrocarbon and olefin interferences, were distinctly dissolved in the reactor to explore the anti-interference capability of the DADES (Figure 5e). The inclusion of toluene and paraxylene led to a decrease in desulfurization efficiency from 96.6% to 86.0% and 81.2%, respectively, indicating that the reaction system was resistant to aromatic hydrocarbons. Nevertheless, desulfurization efficiency dropped dramatically to 19.7% with the addition of cyclohexene, suggesting that olefins severely impact EODS performance. Such results are probably due to competing oxidative reactions among sulfur substrates with the olefins. In conclusion, [Bmim]Cl/BA/0.3AA could show excellent EODS activity for fuel with a low olefin hydrocarbon content. Moreover, the presence of nitrogen-containing substances may inhibit the desulfurization process, thereby affecting clean fuel production and oil quality [46,47,48,49]. Hence, quinoline as a typical basic nitrogen compound and carbazole and indole as representative neutral N-containing substrates were used to prepare model oil with an S-content of 200 mg kg^−1^ and N-content of 100 mg kg^−1^. The EODS reaction was then carried out using different oils to explore the selectivity of [Bmim]Cl/BA/0.3AA. In Figure S7, sulfur removal was 96.4% and 96.6% after adding CBZ and QUI, respectively, indicating that [Bmim]Cl/BA/0.3AA exhibited excellent selectivity for DBT in the above oils. However, the sulfur removal decreased from 96.6% to 40.7% after the addition of IND, which is attributed to the competing extractive and oxidative reactions of sulfur substrates with it [50,51,52,53,54]. Furthermore, the EDS efficiency decreased from 22.6% to 2.2% with the addition of CBZ, suggesting CBZ’s significant competitive extraction with sulfur compounds. Therefore, [Bmim]Cl/BA/0.3AA could achieve deep desulfurization for oil with a low IND.

For industrial applications, it is essential to consider the recyclability and stability of catalysts. After the first reaction, the used [Bmim]Cl/BA/0.3AA could be recycled via drying at 60 °C for 8 h for the next cycle. After being recycled three times without treatment, the deep desulfurization was retained (Figure 5f) with a sulfur removal of 95.6%. However, after four cycles, the sulfur removal decreased to 83.7%, which is attributed to the accumulation of oxidative products in the DADES phase, leading to the shielding of active sites. Additionally, the constant generation of H_2_O in the reaction system also impeded the activity of [Bmim]Cl/BA/0.3AA. The used DADES was regenerated by washing with diethyl ether to remove the accumulated oxidative products and then dried for a new run. It is noteworthy that the regenerated DADES exhibited excellent desulfurization performance. Furthermore, FT-IR and ^1^H NMR analysis of the fresh and regenerated DADES are shown in Figure S8. The prominent characteristic peaks of [Bmim]Cl/BA/0.3AA displayed no significant changes, demonstrating good structural stability. Moreover, compared to the BA-based DES reported in the literature, deep desulfurization was achieved under milder conditions and with a faster reaction rate owing to the synergistic effect of the dual-acid HBDs in the DADES (Table S2).

### 2.4. Possible EODS Reaction Mechanism over [Bmim]Cl/BA/0.3AA

**Figure 6:**
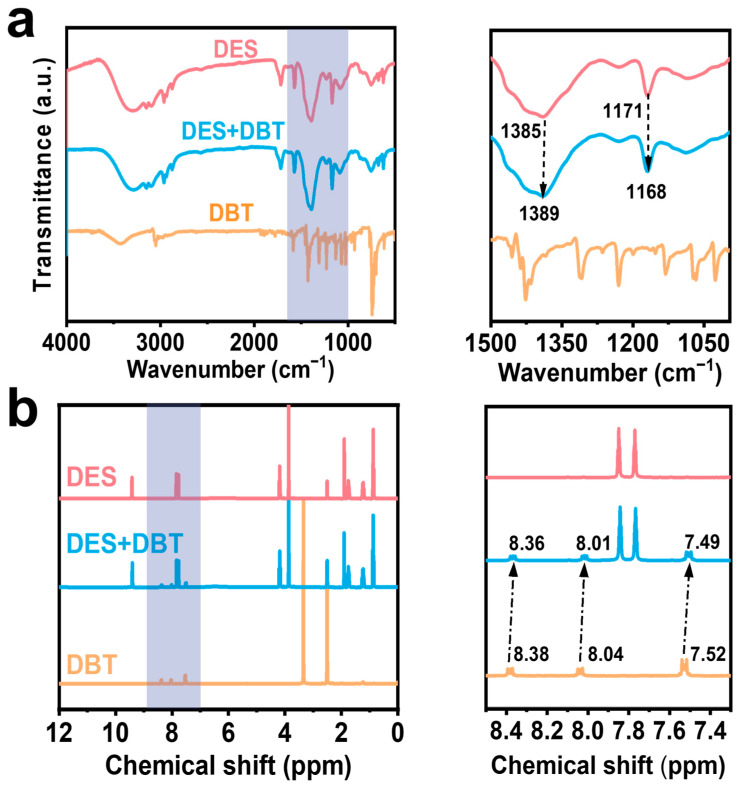
(**a**) FT-IR and (**b**) ^1^H NMR analysis of the interaction of DADES and DBT.

With the intention of investigating the EDS process mechanism, FT-IR was carried out on the mixture of DBT and DADES (molar ratio of 1:0.5). Figure 6a shows that the –COOH blue shifted from 1385 cm^−1^ to 1389 cm^−1^, and the in-plane bending vibration of the C–H bond in the imidazole ring shifted from 1171 cm^−1^ to 1168 cm^−1^. This suggests the presence of weak interactions, including H-bonding interaction and π–π interaction, between the DADES and S-containing compounds [39,42,55]. The observed changes in chemical shifts in the ^1^H NMR spectra further clarify the interaction between DBT and DADES. Figure 6b shows three H signals in the DBT at 8.38, 8.04, and 7.52 ppm, which were shifted to the up-field (8.36, 8.01, and 7.49 ppm) after being added into the DADES. Such results can be attributed to an enhancement in the electron-donating ability of the S atom, which reduced the electron density of H-signals in the benzene ring, improving the accessibility of sulfur compounds to be extracted [55,56].

**Figure 7:**
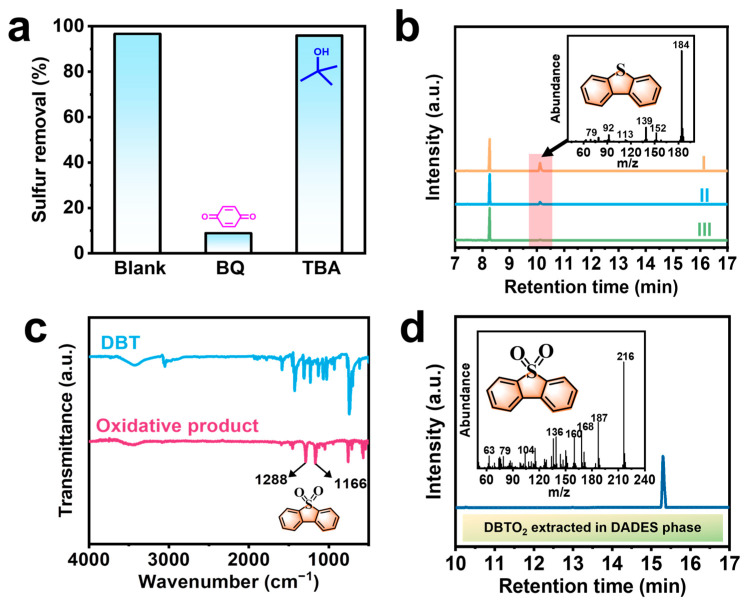
(**a**) Radical quenching experiments. (**b**) GC-MS chromatogram of model oil (I) before the reaction, (II) during, and (III) after the EODS process for 90 min. (**c**) FT-IR spectra of the DBT and corresponding oxidative product. (**d**) GC-MS analysis of the DADES phase (re-extracted by CCl_4_). Experimental conditions: Model oil = 5 mL, O/S = 5, m (DES) = 1.5 g, t = 90 min, T = 40 °C.

The natural mechanism of the ODS process is crucial for designing a novel DADES with excellent desulfurization performance, which has been analyzed by various technologies. According to the active radical capturing experiment (Figure 7a), sulfur removal was barely affected after adding TBA as the scavenger of hydroxyl radicals (•OH) [57,58]. Whereas its catalytic ability was entirely abolished by adding the BQ into the reaction system, inferring that the superoxide (•O_2_^−^) radical might have been the primary active intermediate in the oxidative process [59]. To verify the above conjecture, ESR measurements were conducted using the radical trapping reagent 5,5-dimethyl-1-pyrroline-N-oxide (DMPO). It is notable that after 90 min of the ODS process in the presence of the DADES, a distinctive six-fold peak signal corresponding to the DMPO–•O_2_^−^ was detected (Figure S9) [33], which coincides with the free radical scavenging experiments. The GC-MS chromatogram was analyzed to investigate the EODS process. In Figure 7b, the intensity of the DBT at a retention time of 10.2 min (*m*/*z* = 184) continued to decrease as the reaction progressed [60]. After the reaction, it disappeared after the EODS reaction, which means that sulfur compounds were converted. To deeply explore the oxidative products, diethyl ether was used to extract them. The white solid was separated via a funnel with filter paper and dried at 60 °C for further analysis. In Figure 7c, the characteristic peaks at 1288 cm^−1^ and 1166 cm^−1^ correspond to the S=O bond, which confirms that the oxidative product was DBTO_2_ [61,62]. In combination with the GC-MS analysis (Figure 7d), the high polarity product DBTO_2_ was extracted in the DADES phase, appearing at the retention time of 15.3 min (*m*/*z* = 216) [63]. In the meantime, there was no characteristic peak of the oxidative product in the oil phase, revealing that it was maintained in the DADES phase due to the EDS process. Consequently, it is reasonable to determine that S-containing substrates were eliminated from the model oil after the EODS process.

**Scheme 1:**
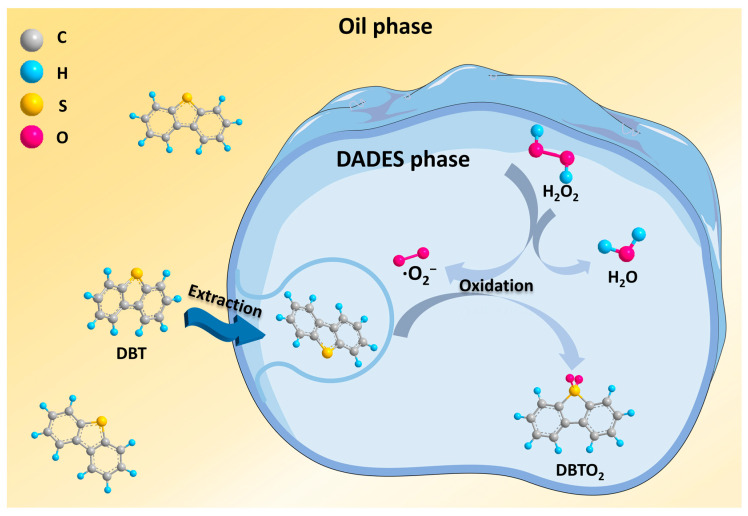
The plausible reaction mechanism of the EODS process by [Bmim]Cl/BA/0.3AA.

Based on the above analyses, a plausible reaction mechanism for the EODS process with [Bmim]Cl/BA/0.3AA is proposed (Scheme 1). Firstly, sulfur compounds were extracted from the oil phase to the DADES phase through their interaction, which promoted sufficient contact among sulfur compounds, oxidants, and active centers. H_2_O_2_ was then dissolved in the DADES phase and catalyzed synergically by the dual-acid HBD, resulting in the formation of •O_2_^−^ radicals with strong oxidizing activity. Subsequently, the S-atom with lone pairs in sulfur compounds was attacked by •O_2_^−^ radicals to form the corresponding sulfone. The oxidative product was then retained in the [Bmim]Cl/BA/0.3AA phase with high solubility, which is convenient for producing clean low-sulfur oil since it is easily separated.

## 3. Experimental Section

### 3.1. Materials

1-Butyl-3-methylimidazolium chloride ([Bmim]Cl, 98%), boric acid (BA, AR), acetic acid (AA, AR), propionic acid (PA, 99.5%), oxalic acid (OA, AR), acetonitrile (ACN, AR), glycolic acid (GA, 98%), dodecane (C_12_H_26_, 98%), and hexadecane (C_16_H_34_, 98%) were brought from Aladdin Industrial Corporation (Los Angels, CA, USA). Polyethylene glycol 200 (PEG-200, C.P.), hydrogen peroxide (H_2_O_2_, 30 wt.%), tetra-chloromethane (CCl_4_, 99.0%), pyridine (AR), methylbenzene (AR), p-xylene (AR), and cyclohexene (99%) were purchased from Sinopharm Chemical Reagent Co., Ltd. (Shanghai, China). Dibenzothiophene (DBT, 98%), 4-methyl dibenzothiophene (4-MDBT, 97%), 4,6-dimethyl dibenzothiophene (4,6-DMDBT, 97%), and ethyl ether (C_4_H_10_O, 98%) were bought from Sigma Aldrich (Burlington, MA, USA). 4-Nitroaniline (99%), *p*-benzoquinone (BQ, 99.5%), and *tert*-butyl alcohol (TBA, 99.5%) were purchased from Shanghai Maclin Biochemical Technology Co., Ltd. (Shanghai, China). All chemicals were at an analytical grade and used directly without further purification.

### 3.2. Preparation of DESs

**Scheme 2:**
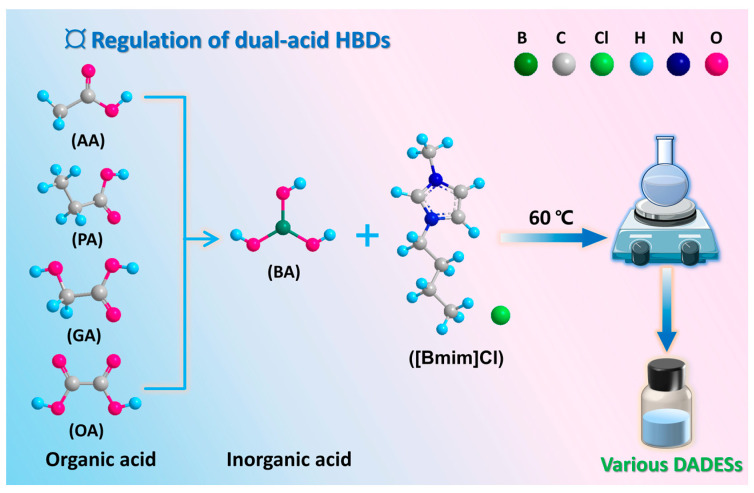
Construction of inorganic–organic dual-acid deep eutectic solvents (DADESs).

A series of new inorganic–organic dual-acid DESs have been fabricated, and the preparation procedure is shown in Scheme 2. Inorganic acid (BA) and different organic acids (AA, PA, GA, OA) were employed as HBDs, and [Bmim]Cl was chosen as the HBA. In the standard procedure, [Bmim]Cl, BA, and AA were added in a round-bottomed flask with a molar ratio of 1:1:*n* (*n* = 0.1, 0.2, 0.3, 0.4, 0.5, 1, 2) for 20 min at 40 °C with vigorous stirring. The produced transparent and homogeneous solvent was designated as [Bmim]Cl/BA/nAA. Similarly, [Bmim]Cl/BA/nPA, [Bmim]Cl/BA/nOA, and [Bmim]Cl/BA/nGA were also synthesized using similar procedures. Additionally, to explore the influence of acidity and viscosity on EODS performance, PEG and ACN were introduced as the third component to prepare additional ternary DES.

### 3.3. Miscibility of DES and Model Oil

To investigate the miscibility of the DES and model oil, the DES and n-dodecane (with equal mass ratios) were mixed in a flask with continuous stirring for 60 min at room temperature. Subsequently, the upper n-dodecane and the lower DADES were separated and characterized by means of FT-IR and ^1^H NMR, respectively. Furthermore, the above experiments were replicated three times to guarantee the accuracy of the data.

### 3.4. Desulfurization Experiment

Model oils were prepared using various sulfur-containing substrates to simulate the environment with different aromatic sulfur compounds in diesel fuel. For instance, DBT was solubilized in n-dodecane to prepare the model oil containing an S-content of 200 mg kg^−1^, while hexadecane was used for the internal standard at a concentration of 4000 mg kg^−1^. Similarly, model fuels with various S-containing substances, including 4-MDBT and 4,6-DMDBT, were prepared using the same procedure. In a typical EODS process, 1.5 g of DES and 5 mL of oil were added to a reactor equipped with a water condenser. The efficiency of the extraction process was tested by stirring at 600 r/min for 15 min at a specified temperature. A specific amount of H_2_O_2_ was dissolved in the reactor to initiate the ODS reaction. At regular periods, the S-content of oil was analyzed by gas chromatography (GC), and the desulfurization capability was determined as Equation (2).

(2) $$Sulfur\;removal\;(\%)=\frac{C_{0}-C_{t}}{C_{0}}\times 100\%$$

## 4. Conclusions

In this work, the construction of DADESs enhanced the mutual solubility of organic and inorganic acids, enabling them to jointly promote the desulfurization process. As expected, DADESs showed superior extraction and catalytic capacity due to the synergistic effect of their dual-acid HBDs, which increased the acidity of the reaction system and reduced the viscosity. Detailed experiments demonstrated the universality of the synergistic effect in different DADESs by adjusting the organic acids, all of which exhibited higher desulfurization capacities than those of binary DES with a single acid HBD. Among them, [Bmim]Cl/BA/0.3AA achieved the best sulfur removal of 96.6% at 40 °C. Moreover, it could be regenerated by washing with diethyl ether, proving its potential for industrial application. The dual-acid strategy created a favorable reaction environment, which involved rationally controlling the acidity and viscosity of the reaction system, effectively enhancing the EODS process.

## Figures and Tables

**Table 1:** Desulfurization performance of raw materials and various DESs.

Entry	Sample	Molar Ratio	Sulfur Removal (%)	
			EDS ^a^	EODS ^b^
1	BA ^c^	–	–	3.6
2	AA ^d^	–	–	2.3
3	[Bmim]Cl ^e^	–	9.5	13.1
4	[Bmim]Cl/BA	1:1	9.8	75.0
5	[Bmim]Cl/AA	1:0.3	24.1	21.9
6	[Bmim]Cl/BA/AA	1:1:0.3	22.0	96.6
8	BA/AA	1:0.3	White solid	

Experimental conditions: Model oil = 5 mL, O/S = 5, m (DES) = 1.5 g, ^a^ t = 15 min, ^b^ t = 90 min, T = 40 °C, ^c^ m (BA) = 0.36 g, ^d^ m (AA) = 0.14 g, ^e^ m ([Bmim]Cl) = 1.0 g.

**Table 2:** Hammett function values of indicator and different DESs.

Entry	Simple	A_max_	[In] (%)	[InH^+^] (%)	*H*_0_
I	4-nitroaniline	1.323	100	–	
II	[Bmim]Cl/BA	1.242	93.88	6.12	2.17
III	[Bmim]Cl/0.3AA	1.230	92.97	7.03	2.11
IV	[Bmim]Cl/BA/0.3AA	1.198	90.55	9.45	1.97

## Data Availability

The data presented in this study are available on request from the corresponding author.

## Supplementary Materials

The following supporting information can be downloaded at: https://www.mdpi.com/article/10.3390/molecules28237743/s1, Characterization and analytical methods; Mutual solubility investigations; Other desulfurization performance results; Figure S1: The mixture of (a) BA/AA and (b) BA/0.3AA after stirring at 60 °C for 20 min; Figure S2: FT-IR (a) and ^1^H NMR (b) spectra of fresh DES and DES phased after mixing; (c) FT-IR and (d) ^1^H NMR spectra of fresh n-dodecane and n-dodecane after mixing; Figure S3. The EDS efficiency of [Bmim]Cl/BA/0.3AA. Experimental conditions: Model oil = 5 mL, m (DES) = 1.5 g, T = 40 °C; Figure S4: ^1^H NMR spectra of BA, AA, and DESs; Figure S5: Viscosity of [Bmim]Cl/BA/0.3AA at different temperatures; Figure S6: The sulfur removal of [Bmim]Cl/BA/0.3AA with different mass concentrations of H_2_O_2_. Experimental conditions: Model oil = 5 mL, T = 40 °C, O/S = 5, m (DES) = 1.5 g; Figure S7: The sulfur removal of [Bmim]Cl/BA/0.3AA with the addition of different Nitrogen-containing substances. Experimental conditions: Model oil = 5 mL, T = 40 °C, O/S = 5, m (DES) = 1.5 g; Figure S8: FT-IR and ^1^H NMR spectra of fresh and regenerated DADES; Figure S9: ESR spectra of active radicals in reaction systems; Table S1: Hammett function (H_0_) values of various DADESs; Table S2: Comparison of the desulfurization performances of [Bmim]Cl/BA/0.3AA and other BA-based DESs reported in the literature.
