# Supplementary material for: Designing Inorganic–Organic Dual-Acid Deep Eutectic Solvents for Synergistically Enhanced Extractive and Oxidative Desulfurization
Source: Molecules. 2023 Nov 24;28(23):7743. doi: 10.3390/molecules28237743 (PMC10707960; doi:10.3390/molecules28237743)
Supplement: Supplementary file 1 [file molecules-28-07743-s001.zip › molecules-2682063-supplementary.pdf]

Figure S1. The mixture of (a) BA/AA and (b) BA/0.3AA after stirring at 60 °C for 20 min.

Figure S2. FT-IR (a) and ^{1}H NMR (b) spectra of fresh DES and DES phased after mixing; (c) FT-IR and (d) ^{1}H NMR spectra of fresh n-dodecane and n-dodecane after mixing.

Figure S3. The EDS efficiency of [Bmim]Cl/BA/0.3AA.

Figure S4. ^{1}H NMR spectra of BA, AA, and DESs.

Figure S5. The viscosity of [Bmim]Cl/BA/0.3AA at different temperatures.

Figure S6. The sulfur removal of [Bmim]Cl/BA/0.3AA with different mass concentrations of H_2O_2.

Figure S7. The sulfur removal of [Bmim]Cl/BA/0.3AA with the addition of different Nitrogen-containing substances.

Figure S8. FT-IR (a) and ^{1}H NMR (b) spectra of fresh and regenerated DADES.

Figure S9. ESR spectra of active radicals in reaction systems.

Table S1. Hammett function (H_0) values of various DADESs.

Table S2. Comparison of the desulfurization performances of [Bmim]Cl/BA/0.3AA and other BA-based DESs reported in previous kinds of literature.

Characterization and analytical methods

The structure and nature of the interaction in as-prepared DESs were analyzed by FT-IR spectroscopy (Nexus 470, Thermo Electron Corporation) and ^1H NMR (AV400, Brucker) with deuterated DMSO as solvent. To determine the acidity of different DESs, UV–visible (UV–vis) spectra were carried out on a UV-2600 spectrophotometer with distilled water reference. The viscosity of as-prepared DESs was detected by the ROTAVISC lo-vi Complete. Initially, the viscometer is levelled, connected to the water bath temperature controller and fitted with temperature control devices. The rotor and vessel containing the DES to be measured are then fitted to the viscometer. Subsequently, stabilize the temperature in the viscometer according to the required testing temperature and adjust the speed to the appropriate value within the measuring range to start the measurement. Finally, the viscosity values on the electronic display are recorded when they have stabilized. After the measurement, the rotor and container are cleaned to measure the following sample. The S content was evaluated by GC (GC-2010 PLUS, Shimadzu), and the column was SH-Rtx-5 (30 m × 0.25 mm × 0.25 μm). The temperature of the injector is set to 250 °C, and the detector is a hydrogen flame ionization detector (FID). The GC oven program was from 100 to 250 °C with a rate of 20 °C/min, and the temperature of GC-FID was set to 300 °C. The retention time of DBT, 4-MDBT, and 4,6-DMDBT on the detector column was 5.89 min, 6.45 min, and 6.44 min, respectively. The oxidative product was analyzed by gas chromatography-mass spectrometry (GC-MS), which was performed on Agilent 7890A-5975C (Agilent Corporation) equipped with an HP-5 (30 m) capillary column. After an ODS reaction, the used DES was dried in a vacuum at 80 °C and re-extracted by tetrachloromethane (CCl_4) to collect the oxidative product. Then, the CCl_4 phase was further detected by GC-MS. Besides, the used DES was washed with Ethyl ether to collect white solids by filtration, which were characterized by FT-IR and ^1H NMR to determine the structure of the oxidation products further. The FT-IR and ^1H NMR spectra of regenerated DES were analyzed to determine

the stability. The active intermediate was determined by Electron spin resonance (ESR) signals on a Bruker EMX PLUS (German), using DMPO as a radical scavenger.

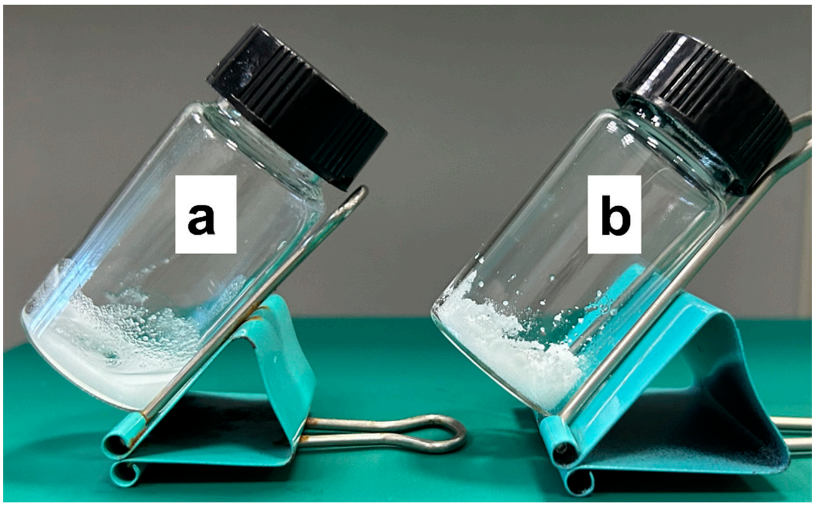

Figure S1. The mixture of (a) BA/AA and (b) BA/0.3AA after stirring at 60 °C for 20 min.

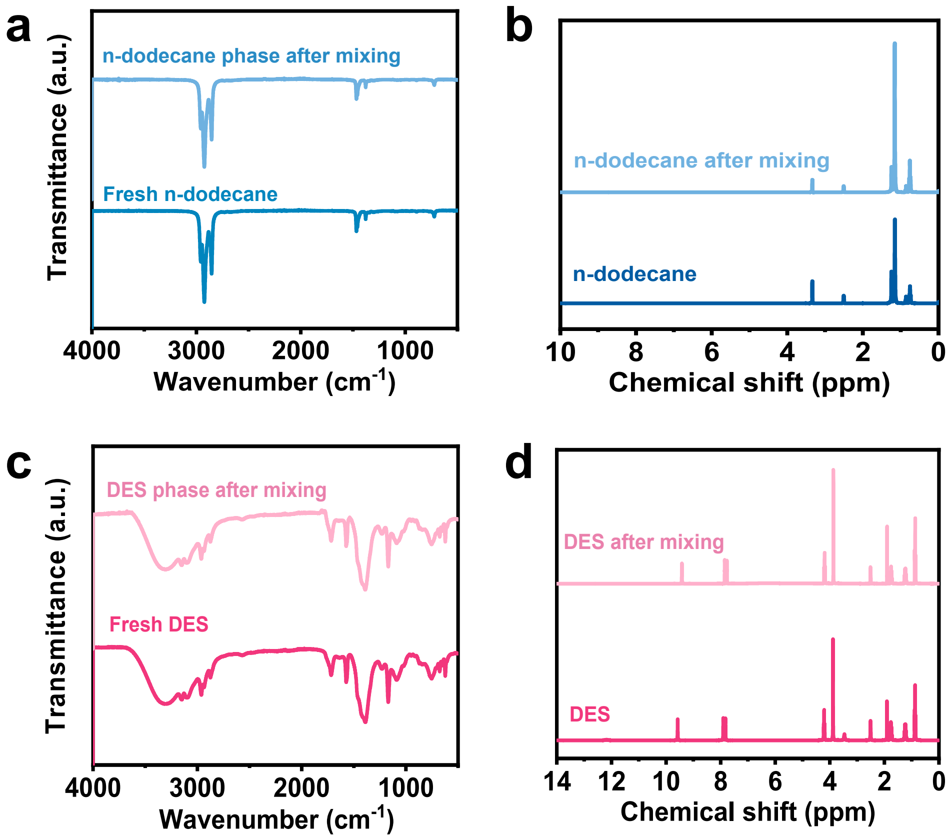

Figure S2. FT-IR (a) and ^{1}H NMR (b) spectra of fresh DES and DES phased after mixing; (c) FT-IR and (d) ^{1}H NMR spectra of fresh n-dodecane and n-dodecane after mixing.

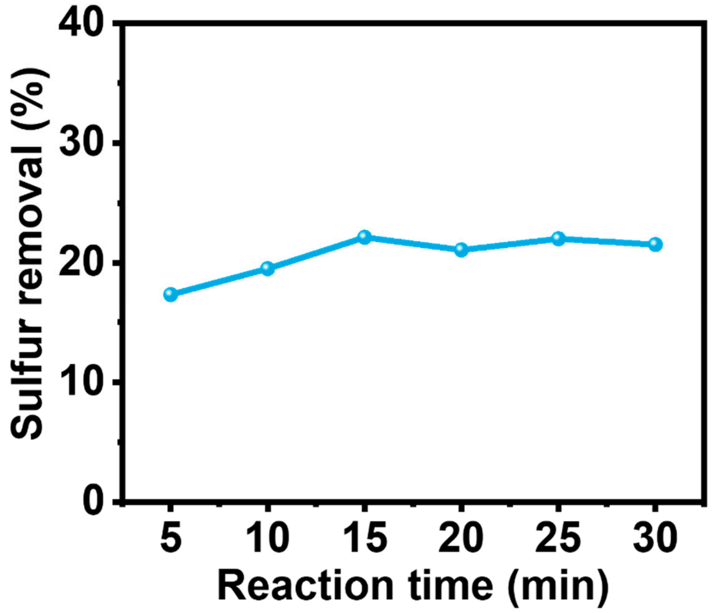

Figure S3. The EDS efficiency of [Bmim]Cl/BA/0.3AA. Experimental conditions: Model oil = 5 mL, m (DES) = 1.5 g, T = 40 °C.

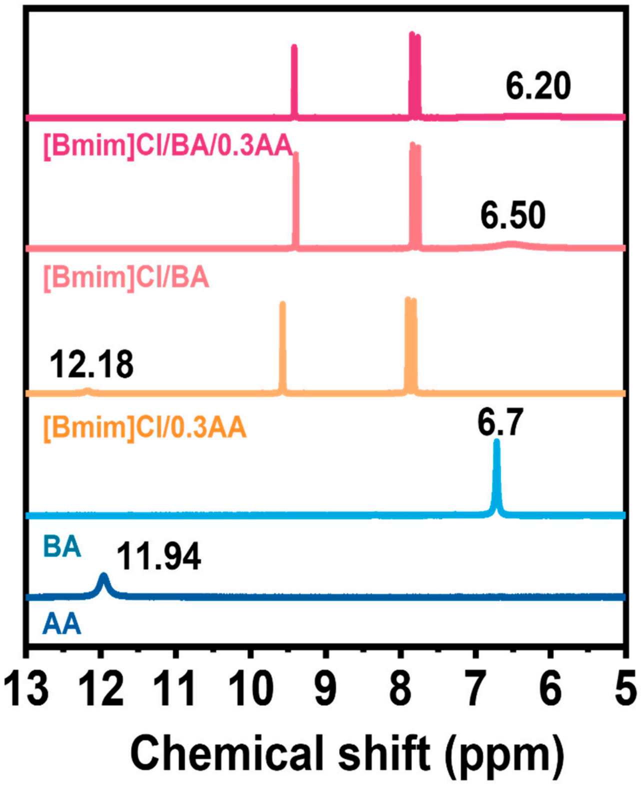

Figure S4. ^{1}H NMR spectra of BA, AA, and different DESs.

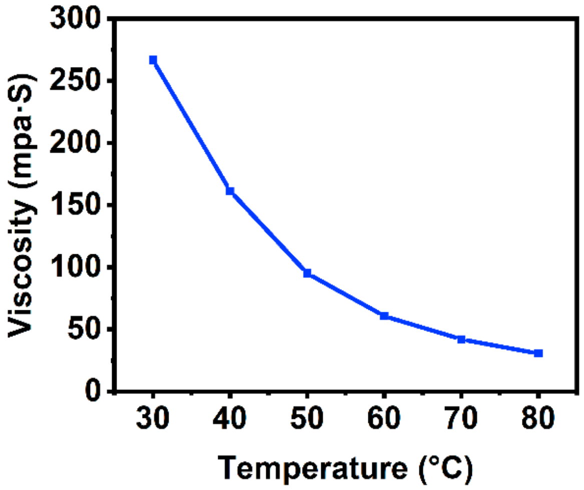

Figure S5. The viscosity of [Bmim]Cl/BA/0.3AA at different temperatures.

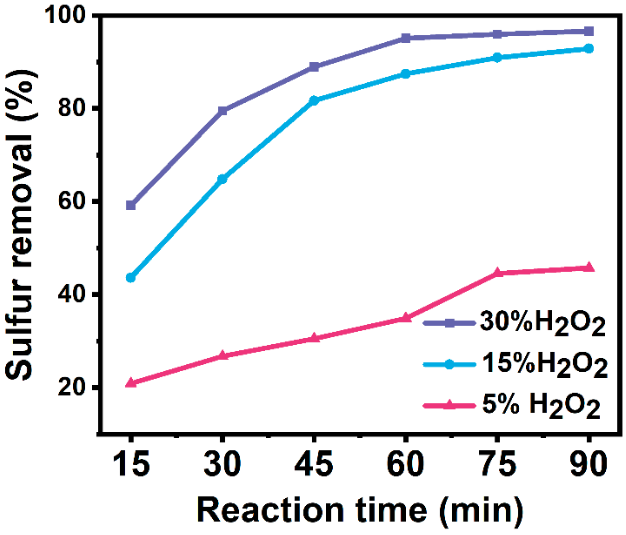

Figure S6. The sulfur removal of [Bmim]Cl/BA/0.3AA with different mass concentrations of H_2O_2. Experimental conditions: Model oil = 5 mL, T = 40 °C, O/S = 5, m (DES) = 1.5 g.

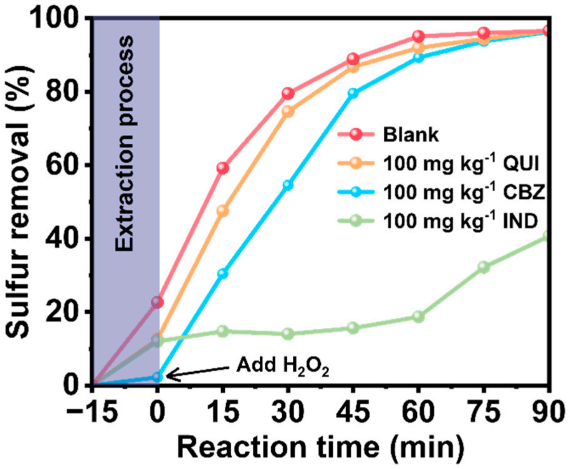

Figure S7. The sulfur removal of [Bmim]Cl/BA/0.3AA with the addition of different Nitrogen-containing substances. Experimental conditions: Model oil = 5 mL, T = 40 °C, O/S = 5, m (DES) = 1.5 g.

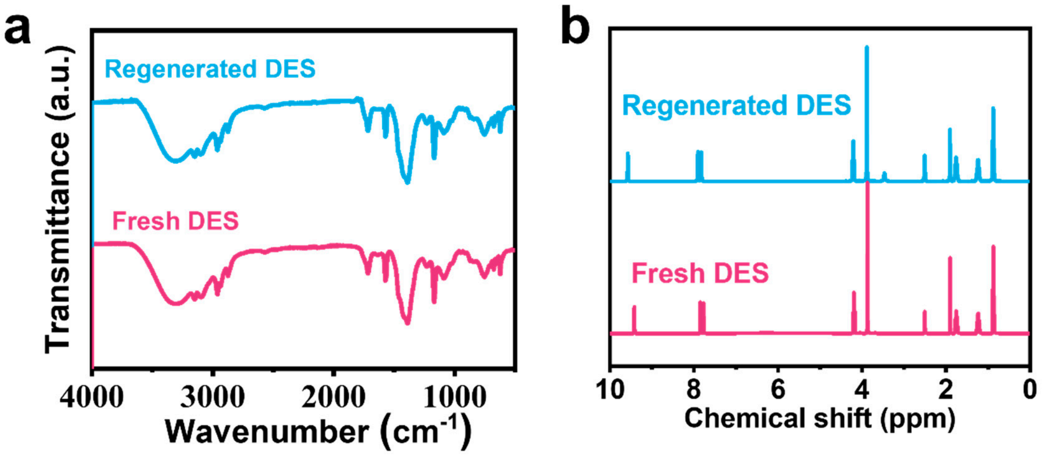

Figure S8. FT-IR (a) and ^{1}H NMR (b) spectra of fresh and regenerated DADES.

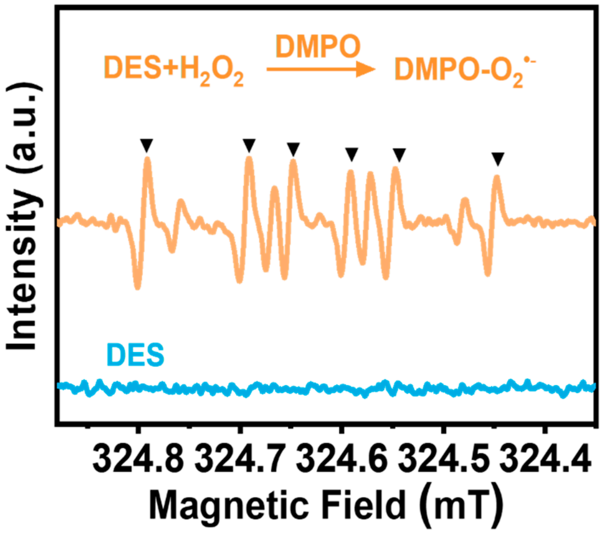

Figure S9. ESR spectra of active radicals in reaction systems.

Table S1. Hammett function values of various DADESs.

Entry	DESs	A_{max}	[In] (%)	$[InH^+]$ (%)	H_0
1	4-nitroaniline	1.14	100	0	/
2	[Bmim]Cl/0.3ACN/BA	1.137	99.74	0.26	3.57
3	[Bmim]Cl/0. 3PEG/BA	1.134	99.47	0.53	3.26
4	[Bmim]Cl/0. 3PA/BA	1.129	99.04	0.96	3.00
5	[Bmim]Cl/0. 3AA/BA	1.128	98.95	1.05	2.96
6	[Bmim]Cl/0.3GA/BA	1.072	94.04	5.96	2.19
7	[Bmim]Cl/0.3OA/BA	0.861	75.53	24.47	1.48

Table S2. Comparison of the desulfurization performances of [Bmim]Cl/BA/0.3AA and other BA-based DESs reported in previous literature.

DESs	T (°C)	O/S	Reaction time (min)	$m_{(DES)}$ (g)	Sulfur removal (%)		Refs.
					EDS[a]	ODS[b]	
ChCl/1.5BA/PEG	60	4	120	2	36.4	96.4	[1]
$ZnCl_2$/2BA/2PEG	60	5	180	2.5	33.7	98.7	[2]
$[C_{12}DMEA]Cl$/3BA/5EG	65	4	180	2	27.8	95.3	[3]
[Bmim]Cl/BA/0.3AA	40	5	90	1.5	22.6	96.6	This work

References

1. W. Jiang, H. Jia, H. Li, L. Zhu, R. Tao, W. Zhu, H. Li, S. Dai, Boric acid-based ternary deep eutectic solvent for extraction and oxidative desulfurization of diesel fuel. Green Chem. 2019,21,3074-3080.

2. L. Xu, H. Jia, D. Zhu, F. Huan, R. Liu, W. Jiang, W. Zhu, H. Li, Hydrogen bonding boosted oxidative desulfurization by $ZnCl_2$/boric acid/polyethylene glycol-based ternary deep eutectic solvents. J. Mol. Liq. 2022,368,120725.

3. W. Jiang, W. Zhu, H. Li, J. Xue, J. Xiong, Y. Chang, H. Liu, Z. Zhao, Fast Oxidative Removal of Refractory Aromatic Sulfur Compounds by a Magnetic Ionic Liquid. Chem. Eng. Technol. 2014,37,36-42.
